# Dysphagia in schizophrenia: pathological mechanisms and treatment recommendations

**DOI:** 10.3389/fpsyt.2024.1448623

**Published:** 2024-09-17

**Authors:** Jiahui Wang, Caifeng Gao, Cuiyuan Fu, Kun Li

**Affiliations:** Shandong Daizhuang Hospital, Jining, Shandong, China

**Keywords:** schizophrenia, dysphagia, screening methods, evaluation, treatment recommendations

## Abstract

Schizophrenia is a chronic, severe, and disabling mental disorder that significantly impacts individuals’ lives. Long-term treatment with antipsychotic drugs, coupled with the complications of the disease itself, increases the risk of dysphagia in patients. These disorders further heighten the likelihood of choking and asphyxia death among this population. This project aims to comprehensively review the pathological mechanisms behind dysphagia in schizophrenia, alongside proposing early screening and evaluation methods. It also suggests treatment recommendations to mitigate the risks and complications associated with dysphagia in these patients.

## 1 Introduction

Schizophrenia is a severe mental disorder with disruptions in perception, thought, emotion, and behavior, along with discoordination in mental activities (1). Beyond the psychiatric spectrum, it is associated with various physical symptoms, including dysphagia. We searched the PubMed and Google Scholar database using a Boolean logic retrieval strategy to find literature on related topics from its inception to June 2024. Research has shown that the prevalence of dysphagia in the general population is 6% (2), while this rate rises to 23% in patients with schizophrenia (3). It is worth noting that the majority of these are hospitalized schizophrenia patients (66.7%), as discovered by Reagan and others (3). Dysphagia can lead to malnutrition and dehydration when its effectiveness is compromised, and its safety issues can result in aspiration pneumonia, choking, and death (4). From 1983 to 1992, mental disorder patients who died from food choking constituted 6.4% of all sudden or unexpected deaths within this group, equating to 14 deaths per 100,000 individuals due to obstructive asphyxia (5). Despite the higher incidence and mortality rates of dysphagia in schizophrenia patients, it has not garnered adequate attention. Hence, this paper aims to systematically explore the pathological mechanisms and management of dysphagia in schizophrenia patients, striving to better prevent and control the occurrence of dysphagia and its complications within this demographic.

## 2 The pathological mechanisms of dysphagia in schizophrenia

**Figure 1:**
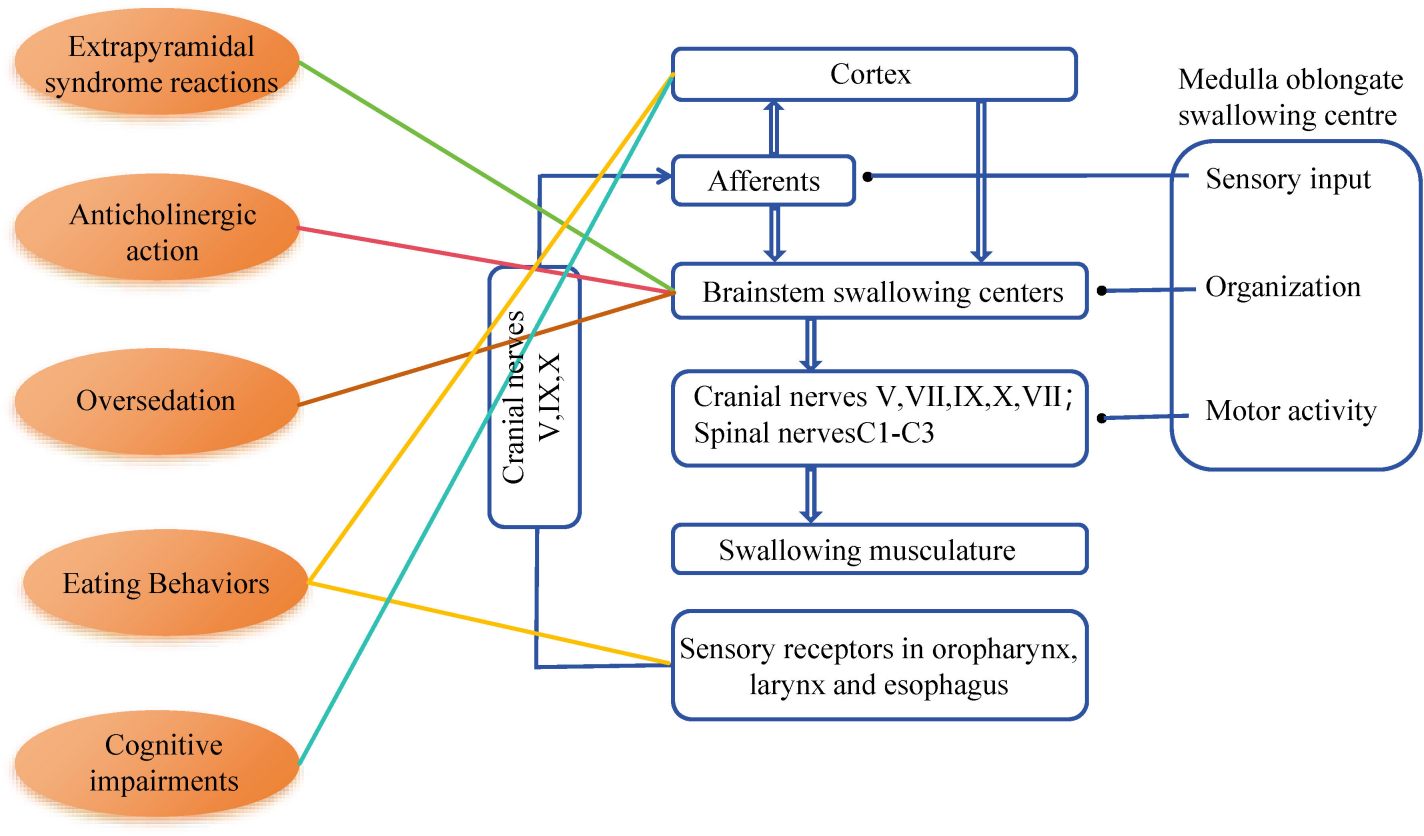
The swallowing network and presumed schizophrenia-related factors for dysphagia. V, trigeminal nerve; VII, facial nerve; IX, glossopharyngeal nerve; X, vagus nerve; XI, accessory nerve.

The primary causes of dysphagia in patients with schizophrenia may be associated with the disease itself and the side effects of related medications. Medication-related side effects include drug-induced Parkinson’s syndrome, acute muscle tension disorder, tardive dyskinesia, dry mouth, drooling, and oversedation (6). Additionally, schizophrenia itself can lead to cognitive dysfunction and eating disorders (see **Figure 1**).

### 2.1 Dysphagia caused by side effects of antipsychotic medications

The preferred method for treating schizophrenia is pharmacotherapy. Most antipsychotic medications work by partially blocking dopamine D2 receptors. However, studies have indicated a correlation between higher doses of antipsychotic medication in hospitalized elderly patients and poorer swallowing function (7). Some antipsychotic drugs may cause extrapyramidal syndrome reactions such as Parkinson’s syndrome, acute laryngeal dystonia (ALD), tardive dyskinesia (TD), and tension disorder, with dysphagia being one of the common adverse reactions to these medications (8). Additionally, the anticholinergic effects and central depressive actions of certain drugs may indirectly affect swallowing function.

#### 2.1.1 Drug-induced Parkinson’s syndrome

Drug-induced Parkinson’s syndrome arises from antipsychotic medications partially blocking the dopaminergic D2 receptors in the nigrostriatal pathway. Its direct effects primarily impact the oral and pharyngeal phases of swallowing. Abnormal tongue motions can lead to sluggish and disorganized bolus production, poor oral bolus control, and slow bolus transport during the oral phase. The delayed swallow reflex, irregular epiglottic movement, delayed, slow, and incomplete laryngeal elevation, poor pharyngeal peristalsis, insufficient glottic protection, pyriform crypt food stagnation, and cricopharyngeal muscle dysfunction are some of the symptoms that can occur during the pharyngeal phase (9). Furthermore, antipsychotic medications may indirectly affect the esophageal phase of swallowing dysfunction: the central anticholinergic action of tranquillizers might interfere with dopamine’s inhibition of inhibitory D2 receptors in the enteric nervous system, which regulates the release of acetylcholine by motor neurons (10). Esophageal dysfunction may result from gastrointestinal motility issues brought on by disruptions in the central dopaminergic pathways. Pneumonia and overflow aspiration are possible consequences of reduced esophageal clearance, particularly when paired with lower esophageal sphincter dysfunction or delayed gastric emptying (11).

Overall, antipsychotic drugs’ direct and indirect anticholinergic actions impair vagal reflexes and alter gastrointestinal motility, which in turn affects how the lower esophageal sphincter, the esophagus, and the stomach operate (12). It’s noteworthy that swallowing abnormalities in Parkinson’s disease patients can be asymptomatic in the early stage (13). Given the similarities in symptoms, signs, and pathophysiology between Parkinson’s disease and drug-induced Parkinson’s syndrome (14), dysphagia caused by the latter may also present without obvious clinical manifestations at onset, making it difficult to promptly recognize. However, most symptoms of drug-induced Parkinson’s syndrome can improve or disappear after adjusting or discontinuing the causative medication (9).

#### 2.1.2 Acute laryngeal dystonia

As a manifestation of acute muscle tension disorder, ALD is an uncommon but possibly fatal side effect of first- and second-generation antipsychotic drugs (15). The most common symptoms and signs of ALD include difficulty breathing, difficulty vocalizing, and wheezing, often accompanied by difficulty swallowing, a sensation of tightness in the throat or feeling choked, and articulation difficulties/speech impairment (15). The impact on swallowing function primarily manifests as oropharyngeal motor dysfunction, poor oropharyngeal clearance, inadequate spontaneous palatal elevation, and a vanished gag reflex (16). For treatment, anticholinergic or antihistamine medications are usually administered via muscle injection or intravenously to rapidly alleviate ALD symptoms and can also be taken orally to prevent recurrence (15).

#### 2.1.3 Tardive dyskinesia

Long-term use of antipsychotic medications can lead to TD, which is an extrapyramidal side effect that is chronic and irreversible. Difficulty swallowing is recognized as a typical feature of TD, often manifesting as involuntary choreiform movements of the tongue, jaw, and lips. Case reports have shown patients with TD exhibiting coordinated movement disorders of oral parts, choreiform movements in the pharyngeal area, and upper esophageal muscle tension disorders (17, 18) (deficiency in upper esophageal function and high tension in the esophageal sphincter). Additionally, severe TD may affect diaphragm movement function, leading to difficulty breathing, wheezing, difficulty swallowing, and a risk of severe choking and aspiration pneumonia (19). After years or decades of exposure to antipsychotic medications, TD usually occurs. Symptoms of TD can persist even after discontinuation or switching medications, making prevention particularly important.

#### 2.1.4 Dry mouth

A meta-analysis showed that the most common anticholinergic side effects during medium to long-term use of typical and atypical antipsychotic medications include blurred vision, dry mouth, and constipation (20). Notably, dry mouth significantly impacts swallowing function and can cause or exacerbate difficulty swallowing. Severe dry mouth affects the formation and propulsion of the bolus (21), thereby increasing the risk of choking. Symptomatic treatments for dry mouth primarily involve increasing fluid intake and consuming moist boluses.

#### 2.1.5 Drooling

The occurrence of drooling seems paradoxical to the anticholinergic action of antipsychotic medications, especially for clozapine, which has the strongest anticholinergic effect, with an incidence rate of drooling between 30% to 80% (22, 23). The pathophysiology of salivary excess associated with clozapine is still unclear, and several mechanisms have been inferred as follows: clozapine is a selective M4 muscarinic receptor agonist and the most anticholinergic antipsychotic medication. It frequently induces salivation (23). Excessive drooling increases the risk of aspiration in patients.

#### 2.1.6 Oversedation

Benzodiazepines, as central nervous system depressants, suppress brainstem-mediated swallowing regulation, selectively inducing pharyngeal swallowing difficulties without reducing the level of consciousness (24). Reduced wakefulness levels caused by sedatives and antiepileptic medications may impair the gag reflex in schizophrenia patients when used alone or in combination with benzodiazepines and/or opioids (25), thereby increasing the risk of coughing and aspiration. Benzodiazepines should be considered a potential reversible cause of pharyngeal swallowing difficulties (24), as their impact on swallowing function can be reversed by reducing the dose or discontinuing the medication.

#### 2.1.7 Abnormal eating behaviors

Antipsychotic medications may induce abnormal eating behaviors such as binge eating, with the pathophysiological mechanism involving the dopaminergic system. Changes in serotonin (5-HT) physiology observed in patients treated with antipsychotic medications affect dopaminergic neurotransmission, leading to the occurrence of abnormal eating behaviors (26).

### 2.2 The impact of the disease itself

Swallowing disorders in schizophrenia are closely related to the cognitive decline and changes in eating behavior caused by the disease itself. The main pathophysiological basis is the reduction in brain cortical and grey matter volume, especially in areas such as the frontal lobe, medial temporal lobe, hippocampus, and amygdala (27–29). Extensive literature confirms a clear association between brain structural abnormalities (such as volume reduction, cortical thinning, and decreased surface area) and cognitive impairments in schizophrenia patients (30–33). The left asymmetrical reduction in the anterior cingulate cortex (34), an important hub for emotional and cognitive control, suggests a possible impairment in cognitive control functions in schizophrenia patients (35, 36).

Furthermore, a study on elderly Japanese requiring long-term care showed that the degree of cognitive impairment is negatively correlated with swallowing function; the more severe the cognitive impairment, the more pronounced the decrease in swallowing ability (37). A substantial amount of moderate-quality evidence indicates that cognitive impairments in schizophrenia patients are primarily in the areas of executive functions and memory (38). Attention not only determines the storage capacity of working memory but also affects the complexity of executive functions (39). Impairment in attention can cause or exacerbate damage to other cognitive functions. These cognitive functions may affect some aspects of eating, such as difficulties in formulating eating plans due to executive function defects, initiating swallowing processes, affecting the amount and speed of eating; decreased attention makes patients easily distracted by external stimuli, leading to reduced eating efficiency, delayed swallow initiation, or piecemeal deglutition; memory disorders make it difficult for patients to learn and remember compensatory swallowing techniques (40, 41).

Therefore, swallowing disorders in schizophrenia patients may be closely related to cognitive impairments caused by the disease itself, necessitating further analysis to clarify the correlation. Recent studies indicate that structural changes in the brains of schizophrenia patients include reductions in gray and white matter volumes, which may affect swallowing control centers and lead to dysphagia. For example, a 2023 study using voxel-based morphometry (VBM) found significant reductions in gray and white matter volumes in schizophrenia patients, particularly in the frontal, temporal, limbic, and parietal lobes (42). These areas are associated with swallowing functions, so these structural changes could explain why some schizophrenia patients experience swallowing difficulties.

In addition to structural changes, disease characteristics such as eating, cognitive, and behavioral disorders can lead to malnutrition (43). A study showed that 16% of schizophrenia patients suffer from binge eating, similar to the abnormal eating behaviors commonly reported by Kulkarni et al. in schizophrenia, which increases the risk of choking and asphyxiation (44). Factors such as impaired chewing skills, diminished attention and focus, hasty eating habits, swallowing large chunks of food, and food residue remaining in the mouth after meals significantly elevate the risk of aspiration among individuals with schizophrenia (3).

There is currently no research on feeding or dietary adjustments for abnormal eating behaviors, but most studies believe that dietary adjustments similar to those for Parkinson’s disease patients are reasonable. To avoid choking, it is recommended to choose foods that are easier to chew, moist, or of lower viscosity. Additionally, researchers suggest using smaller dishes or cups to reduce bolus size or decrease the amount of food on the plate to minimize impulsive eating behaviors. New dining habits, such as putting down utensils while chewing and pacing while eating, help prevent choking. These methods are beneficial for individual dining safety and also reduce dependence on others. It is also advised to adopt the principle of eating smaller, more frequent meals to avoid overeating, inadequate chewing, and bolus swallowing due to excessive hunger.

## 3 Management of dysphagia in patients with schizophrenia

The management of dysphagia in patients with schizophrenia includes screening, assessment, and treatment. Early screening for dysphagia is crucial.

### 3.1 Screening methods

Screening is the recognized first step in managing dysphagia, aiming to identify patients at risk. It is recommended to conduct early screenings for changes in oxygen saturation in all patients at risk of dysphagia to detect aspiration (45, 46). The eating assessment tool-10 (EAT-10) is widely used globally for quickly and conveniently screening populations at high risk for dysphagia, showing good predictive value for dysphagia caused by various diseases (47). It has been demonstrated to be helpful in the screening of swallowing difficulties in a healthy population as well as dysphagia in the oropharyngeal and esophageal phases (48, 49). A high correlation has been shown between the EAT-10 score and the pharyngeal residual, penetration, and aspiration as determined by the videofluoroscopic swallow study (VFSS) and fiberoptic endoscopic assessment of swallowing (FEES) (50, 51). In three minutes, all of a patient’s symptoms can be evaluated (49, 52). And some studies have shown that a cut of 3 is recommended as the best cutoff value for EAT-10 (47). The volume viscosity swallow test, widely used internationally to screen for oropharyngeal dysphagia in patients, is a simple, rapid, and accurate screening tool (53). The test is used to assess the clinical manifestations of impaired swallowing efficacy and impaired safety (53) and also aids medical professionals in choosing the ideal bolus volume and viscosity to maintain each patient’s safe and effective swallow balance. Screening for dysphagia is a systematic and complex process; no single test can provide an accurate screening decision. In clinical practice, healthcare professionals should use a comprehensive set of screening tools tailored to the patient’s condition. Nurses can often be the first to notice symptoms of dysphagia in patients and assist doctors in making a rapid diagnosis. However, there is generally a lack of relevant training in screening and diagnosing dysphagia among psychiatrists and nurses, leading to insufficient attention to patients’ swallowing disorders. Therefore, it is necessary to strengthen the training on screening methods for psychiatrists and nurses to improve their screening capabilities and patient clinical outcomes.

### 3.2 Assessment methods

Assessment is the second step in managing dysphagia. In the event of an unsuccessful swallowing test, the presence of dysphagia poses a need for additional evaluation to elucidate the pathophysiological features of the swallowing impairment.

They are both considered the gold standard for diagnosing dysphagia in clinical settings (54). The VFSS can identify swallowing difficulties, their severity, and characteristics by imaging the swallowing process of different-textured food and/or liquids (55). Through the FEES, anatomical and physiological functions, eating functions, and treatment effects are evaluated. An international survey showed that over 80% of doctors have adopted FEES as a routine diagnostic method for swallowing assessment (56, 57). Both methods are also suitable for assessing swallowing disorders in patients with schizophrenia. However, during the acute phase of mental illness or when certain psychiatric symptoms are present, patients may be unable to cooperate with these examinations.

For high-risk patients unable to undergo instrumental assessments, non-instrumental clinical assessments can meet their needs (58). The majority of research concurs on the following four categories of evaluations, despite the fact that there isn’t yet a thorough summary of clinical assessments (59): 1) cognitive and communication assessment; 2) oral, laryngeal, and pharyngeal anatomical, physiological, and functional assessment (including cranial nerve examination); 3) observation of oral intake and nutritional status during meals; 4) intervention trials (e.g., medication dosage adjustment, posture adjustment, and swallowing action).

As part of a multifaceted evaluation of swallowing issues, patient self-report assessments constitute another non-instrumental clinical assessment. In patient self-assessments, questionnaires are chosen to evaluate the patient’s quality of life, health status, degree of swallowing issues, and even a simple inquiry like “How is swallowing?” can be as effective as detailed screening tools (49).

### 3.3 Treatment recommendations

According to the literature, a multidimensional treatment approach helps better manage dysphagia in patients with schizophrenia, including medication adjustment, swallowing training, and dietary adjustments (60).

#### 3.3.1 Medication adjustment

Medication adjustments may involve switching to another or a second-generation antipsychotic medication, stopping antipsychotic medication treatment, reducing the dosage, or administering medications that can reverse adverse reactions. Reported cases have shown that these methods can positively address swallowing issues, indicating the reversibility of antipsychotic-induced swallowing difficulties (21). Make medication adjustment plans based on different drug side effects. (see **Table 1**).

**Table 1:** Common drug-induced swallowing disorders and their adjustment methods in schizophrenia.

Problem	Common causative agents	medication adjustment
Drug-induced Parkinson’s syndrome	Antipsychotics (FGA and SGA)	• Switch to an antipsychotic with less extensive dopamine blockade (either from a first-generation to a second-generation agent, or to another agent within class) (6) •It is recommended to reduce the dose of orally administered antipsychotics if possible (73) Concomitant use of anticholinergic drugs (biperiden and trihexyphenidyl) or antiparkinsonian drugs (amantadine) (73)
ALD	High potency first-generation neuroleptic agents	• Anticholinergic or antihistamine medications (Intramuscular or intravenous administration)
TD	Antipsychotics (chronic use)	• Reduce dose of antipsychotics (74) • Switch from FGA to SGA (74) • Switch to another SGA (74) • Consider VMAT-2 inhibitors for moderate to severe or disabling TD (75)
Dry mouth	Anticholinergic agents (numerous)	• Increase fluid intake and consuming moist boluses
Drooling	clozapine	• Atropine and tropicamide drops, or oral amitriptyline and amisulpride (76)
Oversedation	benzodiazepines and/or opioids	• Reduce the dose • Discontinue taking the medication.

ALD, Acute laryngeal dystonia; TD, Tardive dyskinesia; FGA, first-generation antipsychotic; SGA, second-generation antipsychotic; VMAT-2, vesicular monoamine transporter-2.

#### 3.3.2 Dietary adjustments

Due to memory impairments or executive function disorders that may affect training outcomes, “active strategies” for dietary adjustments (such as swallowing training) may have limited effects. Therefore, “passive strategies” are recommended, such as choosing soft textures or moist pureed foods to compensate for deficiencies in the oral preparation phase, reducing oral and pharyngeal transport; thickening liquids to reduce aspiration; using taste or temperature to enhance the swallowing reflex (cold drinks are usually easier to swallow); and adjusting eating utensils and bite sizes. It is also recommended to include patients, support personnel, and family members in training to raise awareness of swallowing risks (61).

#### 3.3.3 Non-invasive neurostimulation therapy

A novel therapeutic approach, non-invasive neurostimulation therapy has the benefits of cheap cost, easy operation, and excellent effectiveness. Non-invasive Neurostimulation Therapy represents a “passive strategy” and a novel non-invasive treatment option. A randomized controlled trial found that neuromuscular electrical stimulation (NMES) applied to the sublingual musculature can improve swallowing disorders in Parkinson’s disease patients (62). The therapeutic method of providing stimulation to muscles through surface electrodes is NMES. It is used to promote technology to increase swallowing muscle strength and sensory awareness, and its effect in reducing aspiration may be more significant (62). As a form of peripheral stimulation, NMES therapy is widely used in clinical settings.

**Figure 2:**
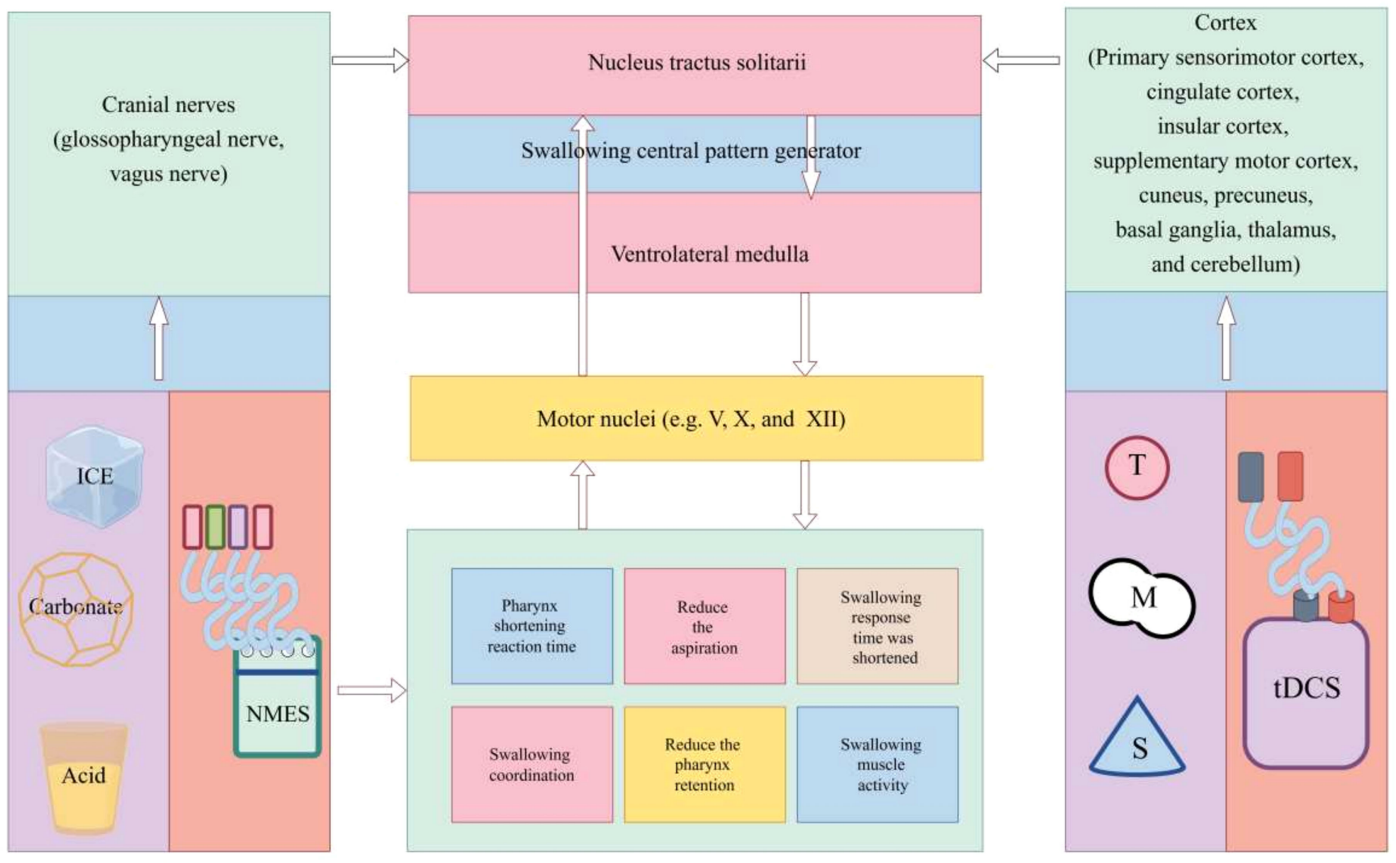
Mechanism of non-invasive neurostimulation therapy for dysphagia. NMES, neuromuscular electrical stimulation; tDCS, transcranial direct current stimulation; rTMS, repetitive transcranial magnetic stimulation.

A meta-analysis revealed that repetitive transcranial magnetic stimulation (rTMS) is effective in helping patients with post-stroke dysphagia improve their ability to swallow (63). When compared to transcranial direct current stimulation (tDCS) and NMES, rTMS appears to be the most efficacious noninvasive neurostimulation therapy for dysphagia following a stroke (64). Depolarization of postsynaptic synapses is the outcome of the former (65). By way of comparison, tDCS is a neuromodulator approach that modifies neural plasticity by direct current (66). Because it specifically stimulates certain brain regions, its use may result in alterations to motor and physiological function (67). By using tDCS, there was a substantial reduction in the risk of aspiration and penetration (68) and oral transit time (69) (see **Figure 2**).

The goal of two neuromodulation methods, tDCS and rTMS, is to modify neuronal excitability throughout the motor cortices’ pharyngeal regions. Both tDCS and rTMS are centrally acting methods that activate pharyngeal cortical areas to initiate neuronal firing and promote neuroplasticity, which are processes of top-down regulation, although having different mechanisms of action (70). By boosting sensory input to pharyngeal cortical regions, NMES, carbonate, ice, and acid stimulation, on the other hand, indirectly boosted motor cortical excitability. This process activates ascending pathways, a bottom-up feedback mechanism (64, 71, 72).

While current physical therapy projects such as tDCS and rTMS are mostly applied to post-stroke dysphagia, there is relatively less research on treating dysphagia in schizophrenia. However, these projects provide valuable information and insights for treating dysphagia, and future research on dysphagia related to schizophrenia can further develop based on this foundation.

## 4 Conclusion and outlook

This paper explores and analyzes the pathophysiological mechanisms of dysphagia in schizophrenia, focusing on swallowing disorders caused by medication adverse reactions, but the research on the impact of the disease itself on swallowing function is not deep and systematic enough. At the same time, dysphagia in schizophrenia has not received sufficient attention; on one hand, there is a lack of effective specific assessment techniques for such patients, and on the other hand, management interventions for their swallowing difficulties are not comprehensive enough. Therefore, this paper reviews the pathophysiological mechanisms, screening and assessment tools of dysphagia in schizophrenia, and provides corresponding dietary and treatment recommendations, hoping to guide and reference clinical practice.
